# Medical Colleges

**Published:** 1873-07

**Authors:** 


*Medical Colleges.*

"The professional readers of the *Companion* cannot but be delighted with its enthusiastic efforts in behalf of medical education. It is a lamentable fact that the subject is almost eschewed by many of the most prominent journals of the day. And yet, it is one which should be held up before the public gaze with unwavering constancy; for, all the good to be expected from practical medicine, in our time and in ages to come, is the result of brain-work. The amount of good accomplished will be in exact proportion to the degree of mental cultivation. Experience, without brain-culture, can not make a man a doctor. Education guides the practitioner into proper estimates of the facts presented, and enables him to profit by experience. Without it, he can never duly appreciate the advantages offered by daily contact with disease. He can never become more than a mere routinist. I am, therefore, more than pleased to see the *Companion* so zealous in the great cause of elevating the standard of medicine through the only possible way—the advance of medical education. Your correspondents, many of them, appear to have caught the cue, and are urging their brethren, through the pages of your excellent journal, to come to the rescue, and save science from the dominion of ignorance. The work is a holy one. Let us pursue it with vigor. Let us fight till we conquer the host of pseudo colleges whose only object is to make money, regardless of consequences—regardless of ethics and integrity—regardless of the ultimate damage to human life consequent upon the multitude of ignorant, stupid and vicious pretenders thrown upon the unsuspecting people—a disgrace to the profession, and an incalculable evil to society. Let us lift up the hands of the good and true men battling in and out of colleges for the benefit of science and a suffering world. If, in your efforts in this direction, you can succeed in opening the eyes of our medical brethren throughout the country, your journal should be justly esteemed their truest friend, and should receive the entire indorsement and support of every true professional gentleman."

The above article was written by one of our most learned friends, and published in the *Companion* last year. We republish it at the request of one of our most useful colaborers, with whom we agree in the opinion that there are colleges in every section of the country that deserve the confidence and patronage of the profession, and these alone should be recommended to medical students by their preceptors, or by the editors of medical journals. While we shall not attempt to correct differences in the management of medical institutions, our readers can rest assured that we will advertise none which have not the men and means of imparting a thorough medical education to students. A legal charter and a suitable or well-constructed building are two, and *only two*, essentials necessary to constitute a first-class medical college.

